# Investigation of the Impact of Atopic Dermatitis (AD) on Stress, Depression, Anxiety, and Suicidal Ideation: A Systematic Review and Meta-Analysis

**DOI:** 10.7759/cureus.63376

**Published:** 2024-06-28

**Authors:** Yaser Mansoor Almutawa, Muneera AlGhareeb, Emma Bhattarai, Jawaher Aljalahma

**Affiliations:** 1 Dermatology and Venereology, Salmaniya Medical Complex, Manama, BHR; 2 College of Medicine and Medical Sciences, Arabian Gulf University, Manama, BHR; 3 Dermatology and Internal Medicine, George Eliot Hospital National Health Service (NHS) Trust, Nuneaton, GBR

**Keywords:** suicidal thoughts, sadness, feeling of fear, tension, atopic eczema

## Abstract

Skin inflammation, known as atopic dermatitis (AD), is often accompanied by various psychological disorders. The objective of this meta-analysis was to assess the impact of AD on stress, depression, anxiety, and suicidal ideation. A comprehensive search was conducted using nine databases. The risk of bias was evaluated using the Newcastle-Ottawa Scale (NOS). ORs were generated to analyze the results. Thirty-one articles met the requirements for inclusion, with 25 deemed of good quality and six of fair quality. A positive association was observed between AD and stress (OR = 1.546; 95% CI: 1.445-1.653; p = 0.000), depression (OR = 1.140; 95% CI: 1.127-1.153; p = 0.000), anxiety (OR = 1.080; 95% CI: 1.063-1.097; p = 0.000), and suicidal ideation (OR = 1.158; 95% CI: 1.144-1.172; p = 0.000). Interestingly, a significant publication bias was found in the outcomes related to depression and anxiety. This analysis suggests that AD significantly impacts the psychological well-being of patients. Stress, depression, anxiety, and suicidal ideation are among the mental health issues commonly associated with AD. Therefore, clinicians should consider mental health evaluations for patients with AD.

## Introduction and background

Atopic dermatitis (AD) is considered one of the most commonly occurring and debilitating skin diseases, especially affecting the pediatric community. Globally, 15-30% of the pediatric population and 10% of adults are reported to be affected by the disease [1,2]. AD typically manifests during childhood or adolescence, which is recognized as a critical period of life marked by metabolic, mental, and physical development. However, the incidence varies among countries [3]. During the 21st century, the one-year incidence of AD varied between 1.2% in Asia and 17.1% in Europe for adults and between 0.96% and 22.6% for children [4].

The pathogenesis of AD, a chronic inflammatory illness, involves the upregulation of the Type II immune mechanism through the activation of Type II helper T cells. This disease particularly leads to impaired colonization of the normal skin flora, such as* Staphylococcus aureus* [5]. Patients may exhibit severe, moderate, or mild forms of AD, depending on the presentation of skin lesions. These lesions are characterized by profound and persistent pruritus that may lead to sleep disorders and psychological issues, including depression and anxiety, ultimately impacting the individual’s quality of life (QoL) [6]. In cases of moderate to severe AD, topical treatments are often less effective, while systemic medications may carry significant toxicity [7].

The notion that skin homeostasis is disrupted in AD is reinforced by the identification of Filaggrin polymorphisms as key genetic factors in AD development, along with epigenetic regulation and other genes mainly involved in the immune system and extracellular matrix [8]. Persistent and recurrent lesions, dry skin, pruritus, and early illness initiation commonly characterize AD. However, atypical morphologies such as prurigo nodularis and follicular/papular dermatitis may also occur [9].

Epidemiological studies have examined the link between psychological stress and AD, revealing that stress exacerbates the condition. Additionally, patients with AD are reported to experience higher levels of psychological stress compared to those without AD [10].

Disrupted sleep patterns, fatigue, social isolation, systemic treatments, and poor QoL may increase the likelihood of depression and anxiety among individuals with AD. Both depression and anxiety are commonly observed in AD patients, possibly due to shared pathological triggers and mechanisms [11,12]. A study conducted in 2017 on the Global Burden of Disease reported that depression has affected more than 300 million people globally and has been identified as the third cause of nonfatal health loss [13-16]. It was estimated that depression alone accounts for around 800,000 fatal suicide attempts worldwide each year [14]. These associated psychological concerns and systemic medication create a daunting situation for both children and adolescents with AD. It has been observed that most AD patients experience depression and anxiety compared to their peers [17].

Several studies have reported increased suicidal tendencies among AD patients [18-20]. However, it is now understood that depression and anxiety are commonly associated with AD [21].

Despite these studies, the true extent and precise pathogenesis of psychological involvement in AD patients remain unclear. Hence, we performed this work, constituting the most updated meta-analysis, to assess the association between AD and several psychiatric comorbidities, including stress, depression, anxiety, and suicidal ideation.

## Review

Material and methods

This study was performed following the recommendations of the Preferred Reporting Items for Systematic Reviews and Meta-Analyses (PRISMA) guidelines [22]. It was registered in the International Prospective Register of Systematic Reviews (PROSPERO) under the number CRD42023405749.

Literature Search

PubMed/Medline, the Cochrane Library, Scopus, Embase, Google Scholar, Web of Science, PsycINFO, and CINAHL were searched by two independent researchers from database inception until March 2024. They used these search terms: “atopic eczema” OR “atopic dermatitis” AND “stress” OR “depression” OR “anxiety” OR “suicidal ideation” OR “suicidality” OR “suicide” OR ‘‘psychosocial’’ OR ‘‘psychological’’ OR “psychiatric”. Two researchers individually carried out each retrieval operation.

Selection of Relevant Articles

After duplicates were eliminated, relevant publications were carefully examined based on their titles and abstracts. Studies that compared patients with and without AD regarding stress, depression, anxiety, and suicidal ideation outcomes were included. The full texts of these studies were then examined to confirm eligibility.

The following requirements must be met for studies to be considered: (1) English language articles; (2) cohort or cross-sectional studies; (3) publications containing original research; (4) assessment of the presence of stress, depression, anxiety, or suicidal thoughts in patients with AD; (5) availability of OR values with a 95% CI. Excluded studies are those that do not fulfill the following requirements: (1) articles without a complete electronic text; (2) non-English published articles; (3) limited outcome data; (4) articles from predatory journals; and (5) editorials, letters, comments, protocols, review papers, and guidelines.

Extraction of Data

Information was gathered from relevant papers by two impartial reviewers in compliance with the inclusion and exclusion criteria. The information was then recorded on an Excel sheet, comprising study number, study and year of publication, study design, country, sample size, gender, age of the participant, year mean ± SD, outcomes and measures, and quality score.

Risk of Bias Assessment

We evaluated the risk of bias in the included articles using the Newcastle-Ottawa Scale (NOS). This tool included three groups: selection bias, comparability of exposed and controlled individuals, and outcome evaluation. Every group received a star rating of either 0 or 1. The total star rating ranged from 0 to 9 stars for cohort research and from 0 to 10 stars for cross-sectional research [23].

Three groups are evaluated by the NOS tool: (1) study group selection (up to four and five stars for cohort and cross-sectional studies, respectively); (2) study group comparability (up to two stars); and (3) outcome evaluation (up to three stars). Two authors separately assessed the quality, and disputes were settled by discussion. Research that receives seven, nine, or ten stars is considered high-quality; research that receives four to six stars is considered fair-quality; and research that receives zero to three stars is considered low-quality [23].

Statistical Analysis

The statistical analyses were performed based on the Comprehensive Meta-Analysis version 3.7 (Biostat Inc., USA). ORs with 95% CIs were estimated to assess the outcomes. A p-value less than 0.05 is typically considered to be statistically significant. Article heterogeneity was assessed using the Cochrane chi-squared test, I^2^, and p-values. I^2^ values ≥50% and P < 0.05 showed a moderate to high degree of heterogeneity. In this case, a random-effects model was used. I^2^ values <50% and p > 0.05 revealed low heterogeneity, and a fixed-effects model was adopted [24]. Funnel plot asymmetry and Egger’s test were analyzed to estimate publication bias.

Results

Selection and Characteristics of Relevant Articles

**Figure 1:**
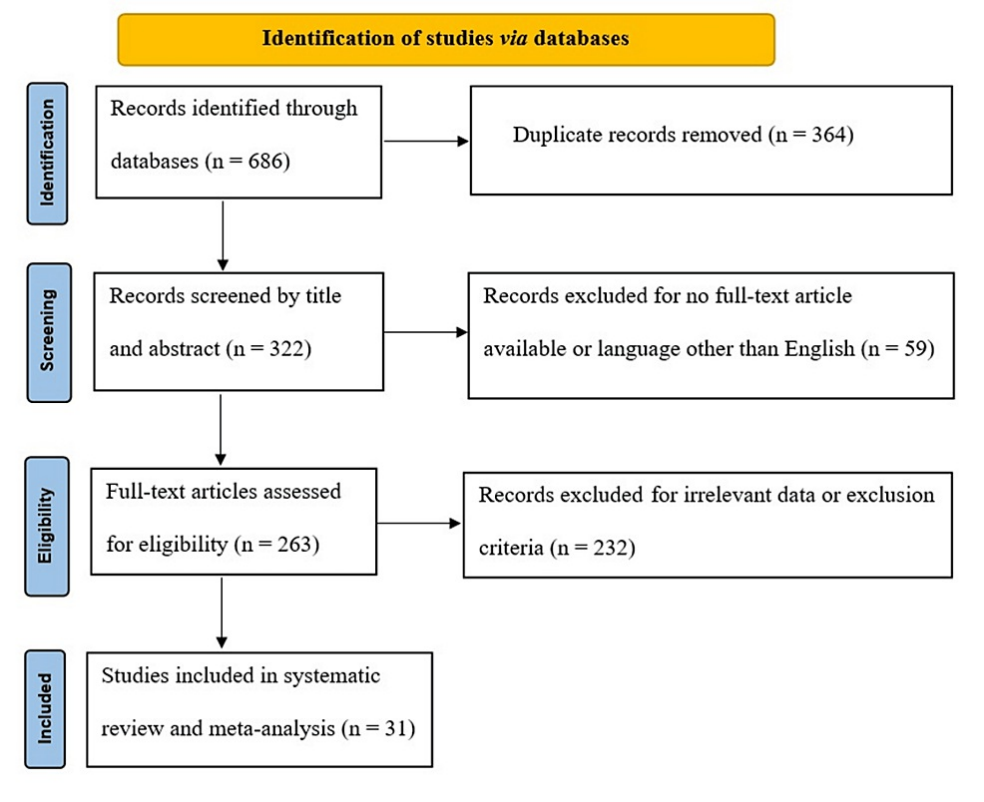
PRISMA flow diagram PRISMA, Preferred Reporting Items for Systematic Reviews and Meta-Analyses

After screening the 686 studies that we found in the databases, 263 abstracts were deemed potentially eligible and subsequently retrieved for full-text analysis. Thirty-one publications met all the criteria and were included in this study. Figure 1 depicts the flow diagram for the literature search.

They were distributed across 12 countries and were published between 2011 and 2023. This work included 26 cross-sectional studies and five observational studies. The case group comprised 20 to 600,125 participants, while the control group ranged from 26 to 2,341,285,705 participants. Twenty-five studies investigated the outcomes of depression, 19 studies examined anxiety, and only four studies examined stress. However, 11 research studies explored suicidal ideation. Overall, the included research received ratings between five and eight stars. Twenty-five of the studies were deemed of good quality, with six papers rated as fair quality (Table 1).

**Table 1:** Features of the studies AD: atopic dermatitis; CES-D: Center for Epidemiologic Studies Depression Scale; CDI-S: Children’s Depression Inventory-Short Form; DASS-42: Depression Anxiety Stress Scale-42; ECI-4: Early Childhood Inventory 4; GAD-7: Generalized Anxiety Disorder-7; HADS: Hospital Anxiety and Depression Scale; HSCL-10: Hopkins Symptom Checklist-10; K6: Kessler psychological distress score; PHQ2: Patient Health Questionnaire; SN: Study Number; NOS: Newcastle-Ottawa Scale

SN	Study and year of publication	Design of study	Country	Size of the sample		Gender		Participants age, years (mean ± SD)		Outcomes and measures	NOS
				AD	Non-AD	Male	Female	AD	Non-AD		
1	Ahn et al. (2019) [25]	Cross-sectional	Republic of Korea	42,641	139,486	103,938	78,189	Multi-age groups		Depression, anxiety, and suicidal ideation	8
2	Augustin et al. (2015) [26]	Cross-sectional	Germany	30,354	262,827	150,244	142,937	≤18		Depression	6
3	Bahreinian et al. (2011) [27]	Cross-sectional	Canada	20	411	243	188	12.5		Depression (CDI-S)	7
4	Catal et al. (2016) [28]	Cross-sectional	Turkey	80	74	79	75	48.40 ± 15.70	49.90 ± 15.19	Anxiety (ECI-4)	8
5	Cheng and Silverberg (2019) [29]	Cross-sectional	USA	436,918	2.34E + 09	1.07E + 09	1.27E + 09	≥18		Depression (PHQ2) (K6)	7
6	Cheng et al. (2015) [30]	Cohort	Taiwan	8,208	8,208	6,544	9,872	32.60 ± 16.06		Depression and anxiety	7
7	Dalgrad et al. (2015) [31]	Cross-sectional	Multi-countries	162	4,832	2,046	2,948	47.20 ± 17.90	41.10 ± 13.60	Depression (HADS), anxiety (HADS), and suicidal ideation	6
8	Dieris-Hirche et al. (2017) [32]	Cross-sectional	Germany	181	64	67	178	27.60 ± 8.30	29.70 ± 10.00	Depression (HADS), anxiety (HADS), and suicidal ideation (Pöldinger’s scale)	8
9	Eckert et al. (2017) [33]	Cross-sectional	USA	349	698	332	715	45.80 ± 14.90	46.30 ± 15.50	Depression and anxiety	8
10	Halvorsen et al. (2014) [34]	Cross-sectional	Norway	346	3,210	1,592	1,964	18-19		Suicidal ideation (HSCL-10)	6
11	Hon et al. (2014) [35]	Cross-sectional	China	120	26	85	61	16	16	Depression (DASS-42), anxiety (DASS-42), and stress (DASS-42)	7
12	Huang et al. (2021) [36]	Cohort	USA	86,969	116,564	105,307	98,226	5.30 ± 5.10	8.70 ± 5.20	Anxiety	7
13	Iannone et al. (2022) [37]	Cohort	Italy	32	91	60	63	46 ± 19		Depression and anxiety	6
14	Kang et al. (2023) [38]	Cross-sectional	Republic of Korea	71,434	221,057	146,922	145,569	15.01 ± 1.75		Depression and suicidal ideation	7
15	Kim et al. (2015) [39]	Cross-sectional	Republic of Korea	434	23,008	9,454	13,988	39.69 ± 15.56	48.71 ± 16.54	Depression	7
16	Kim et al. (2015) [40]	Cross-sectional	Republic of Korea	1,517	118,991	120,508	0	19.80 ± 1.00	20.00 ± 1.20	Depression and anxiety	6
17	Kwak and Kim (2017) [41]	Cross-sectional	Republic of Korea	157	11,756	5,877	6,036	35.20 ± 1.30	45.30 ± 0.30	Depression, stress, and suicidal ideation	7
18	Kye and Park (2017) [42]	Cross-sectional	Republic of Korea	237	19,362	8,461	11,138	50.60 ± 0.12		Suicidal ideation	5
19	Kyung et al. (2020) [43]	Cross-sectional	Republic of Korea	15,536	46,740	31,624	30,652	12-18		Depression, stress, and suicidal ideation	8
20	Kyung et al. (2020) [44]	Cross-sectional	Republic of Korea	173,692	614,719	406,597	381,814	13-18		Suicidal ideation	8
21	Lee and Shin (2017) [45]	Cross-sectional	Republic of Korea	4,904	67,531	36,655	35,780	12-17		Depression and suicidal ideation	8
22	Mizara et al. (2012) [46]	Cross-sectional	UK	54	53	43	64	34.70 ± 12.10	31.40 ± 7.40	Depression and anxiety	7
23	Park et al. (2023) [47]	Cross-sectional	Republic of Korea	600,125	2,072,045	1,387,479	1,284,690	14.97 ± 1.78		Depression and stress	7
24	Silverberg et al. (2019) [48]	Cross-sectional	USA	602	2,291	1,342	1,551	46.6		Depression (HADS) and anxiety (HADS)	8
25	Thyssen et al. (2018) [18]	Cohort	Denmark	1,044	8,612	4,453	5,203	52.5		Depression and anxiety	7
26	Treudler et al. (2020) [49]	Cross-sectional	Germany	372	9,109	4,077	5,404	52	58	Depression (CES-D) and anxiety (GAD-7)	7
27	Vittrup et al. (2021) [50]	Cross-sectional	Denmark	14,283	142,830	89,518	67,595	1.9		Depression and anxiety	8
28	Wan et al. (2023) [51]	Cohort	USA	409,431	1,809,029	1,148,110	1,070,350	5.5		Depression, anxiety, and-suicidal ideation	7
29	Whiteley et al. (2016) [52]	Cross-sectional	USA	428	74,572	36,289	38,711	44.30 ± 0.80	46.60 ± 0.02	Depression and anxiety	7
30	Yaghmaie et al. (2013) [53]	Cross-sectional	USA	10,401	69,095	41,315	38,181	<18		Depression and anxiety	8
31	Zhang et al. (2023) [54]	Cross-sectional	The Netherlands	5,196	51,174	22,577	34,319	55.8 ± 12.2		Depression and anxiety	8
Total		Cross-sectional: 26; cohort: 5	Republic of Korea: 10; Germany: 3; Canada: 1; Turkey: 1; USA: 7; Taiwan: 1; Multi-countries: 1; Norway: 1; China: 1; Italy: 1; UK: 1; Denmark: 2; The Netherlands: 1	1,916,227	2.35E + 09	1.07E + 09	1.28E + 09	-			

Outcomes

**Figure 2:**
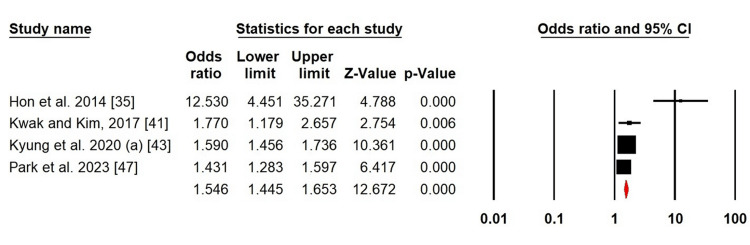
Forest plot of pooled ORs for stress Hon et al. (2014) [35]; Kwak and Kim (2017) [41]; Kyung et al. (2020a) [43]; Park et al. (2023) [47]

Stress: Because of the high level of heterogeneity of stress in patients with AD, we adopted a random-effects design (χ^2^ = 18.43, p = 0.000, I^2^ = 83.72%). In patients with AD, the forest plot analysis revealed a greater risk of stress (OR = 1.546; 95% CI: 1.445-1.653; p = 0.000) (Figure 2).

**Figure 3:**
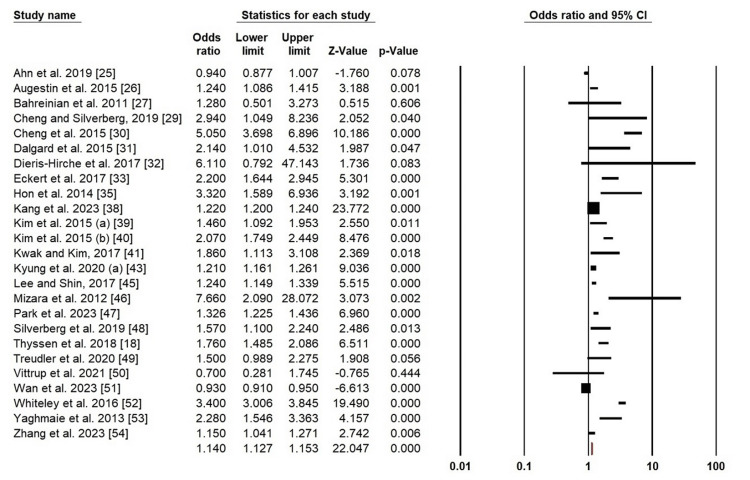
Forest plot of pooled ORs for depression Ahn et al. (2019) [25]; Augustin et al. (2015) [26]; Bahreinian et al. (2011) [27]; Cheng and Silverberg (2019) [29]; Cheng et al. (2015) [30]; Dalgrad et al. (2015) [31]; Dieris-Hirche et al. (2017) [32]; Eckert et al. (2017) [33]; Hon et al. (2014) [35]; Kang et al. (2023) [38]; Kim et al. (2015a) [39]; Kim et al. (2015b) [40]; Kwak and Kim (2017) [41]; Kyung et al. (2020a) [43]; Lee and Shin (2017) [45]; Mizara et al. (2012) [46]; Park et al. (2023) [47]; Silverberg et al. (2019) [48]; Thyssen et al. (2018) [18]; Treudler et al. (2020) [49]; Vittrup et al. (2021) [50]; Wan et al. (2023) [51]; Whiteley et al. (2016) [52]; Yaghmaie et al. (2013) [53]; Zhang et al. (2023) [54]

Depression: Due to the high level of heterogeneity of depression in AD patients, we used a random-effects model (χ^2^ = 1000.871, p = 0.000, I^2^ = 97.60%). A higher risk of depression has been identified in the forest plot analysis in patients with AD (OR = 1.140; 95% CI: 1.127-1.153; p = 0.000) (Figure 3).

**Figure 4:**
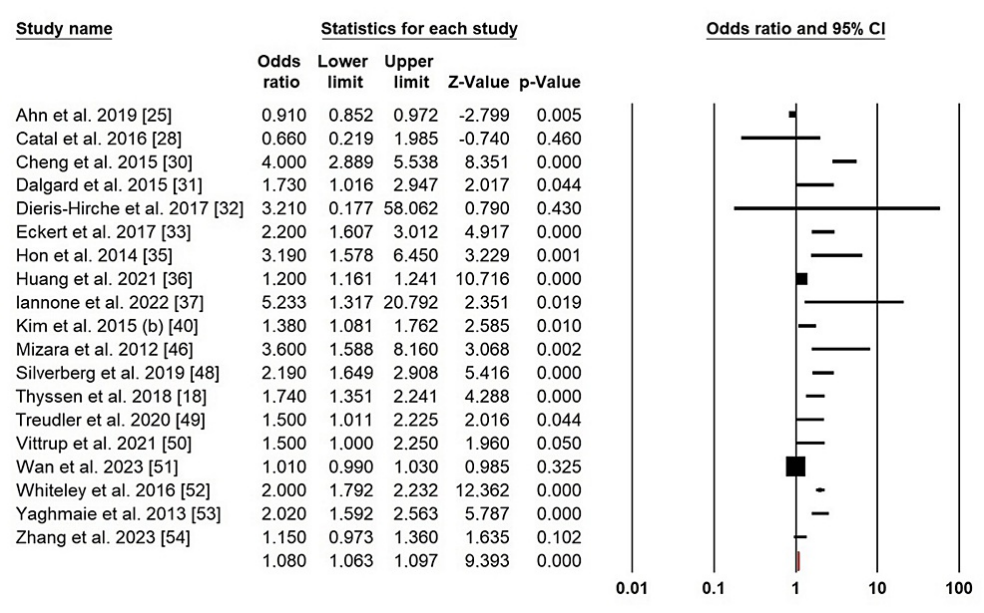
Forest plot of pooled ORs for anxiety Ahn et al. (2019) [25]; Catal et al. (2016) [28]; Cheng et al. (2015) [30]; Dalgrad et al. (2015) [31]; Dieris-Hirche et al. (2017) [32]; Eckert et al. (2017) [33]; Hon et al. (2014) [35]; Huang et al. (2021) [36]; Iannone et al. (2022) [37]; Kim et al. (2015b) [40]; Mizara et al. (2012) [46], Silverberg et al. (2019) [48]; Thyssen et al. (2018) [18]; Treudler et al. (2020) [49]; Vittrup et al. (2021) [50]; Wan et al. (2023) [51]; Whiteley et al. (2016) [52]; Yaghmaie et al. (2013) [53]; Zhang et al. (2023) [54]

Anxiety: Given the high level of heterogeneity of anxiety in AD patients, we implemented a random-effects model (χ2 = 411.236, p = 0.000, I^2^ = 95.62%). The forest plot analysis of the patients with AD showed an elevated risk of anxiety (OR = 1.080; 95% CI: 1.063-1.097; p = 0.000) (Figure 4).

**Figure 5:**
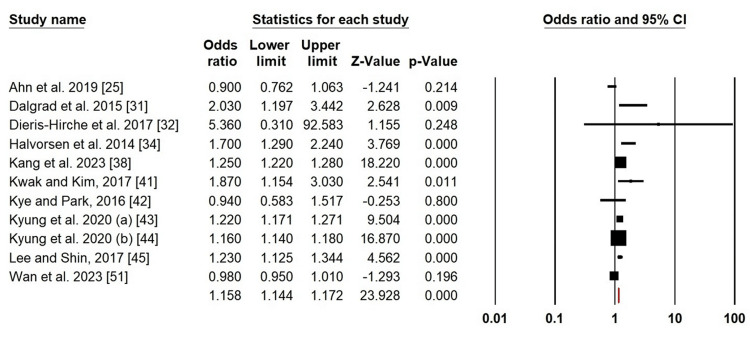
Forest plot of pooled ORs for suicidal ideation Ahn et al. (2019) [25]; Dalgrad et al. (2015) [31]; Dieris-Hirche et al. (2017) [32]; Halvorsen et al. (2014) [34]; Kang et al. (2023) [38]; Kwak and Kim (2017) [41]; Kye and Park (2016) [42]; Kyung et al. (2020a) [43]; Kyung et al. (2020b) [44]; Lee and Shin (2017) [45]; Wan et al. (2023) [51]

Suicidal ideation: Owing to the significant heterogeneity of suicidal ideation in patients with AD, we applied a random-effects design (χ2 = 187.319, p = 0.000, I^2^ = 94.66%). In AD patients, the forest plot analysis revealed a heightened risk of suicidal ideation (OR = 1.158; 95% CI: 1.144-1.172; p = 0.000) (Figure 5).

Publication Bias

**Figure 6:**
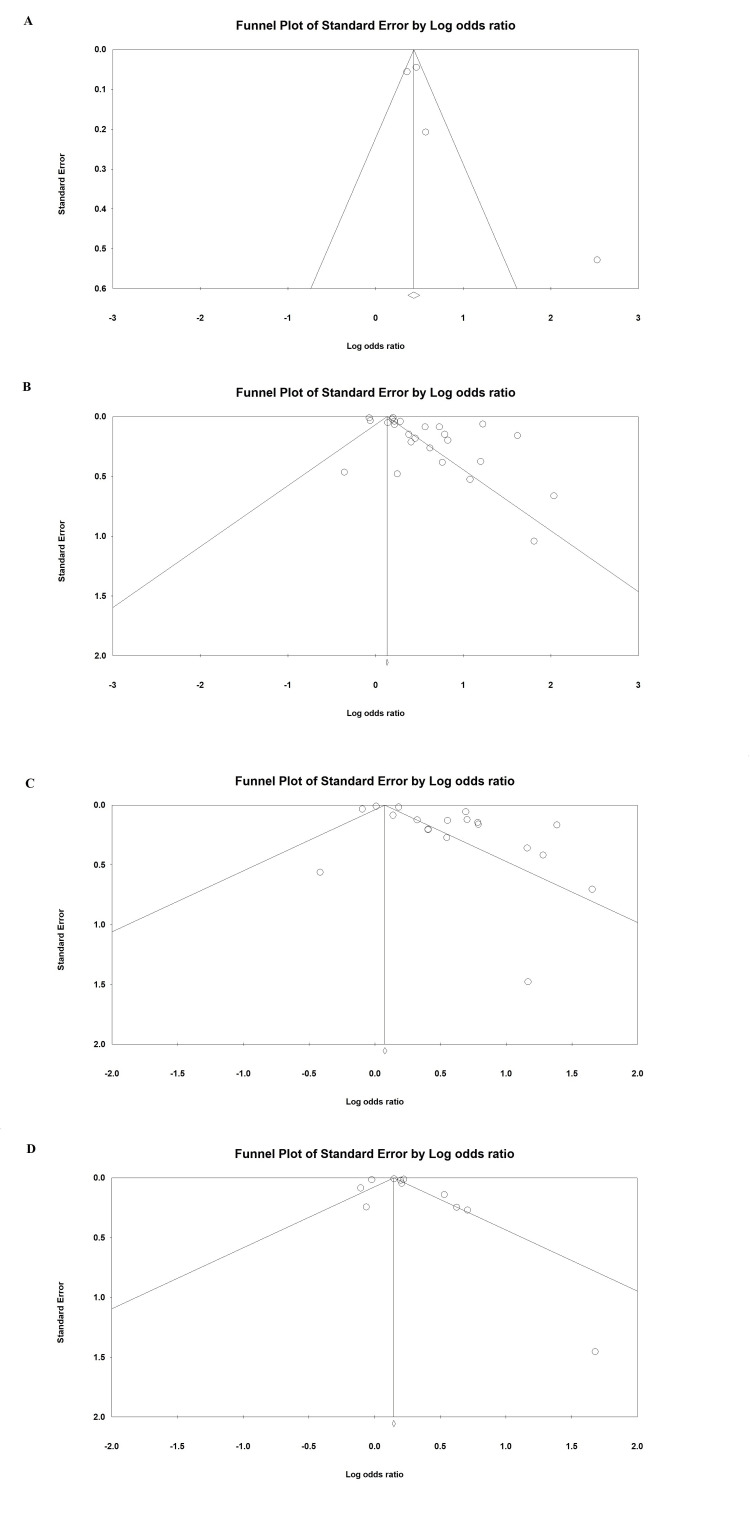
The funnel plots of the included articles reveal no evidence of publication bias for stress (A) and suicidal ideation (D) outcomes; however, they do indicate evidence of publication bias for depression (B) and anxiety (C) outcomes The horizontal axis displays the association’s size, and the vertical axis displays the standard error. The calculated 95% confidence range for the standard error is represented by the two lines on either side of the vertical line, which is the fixed effects summary estimate.

Egger’s test and funnel plot demonstrated no publication bias for stress (p = 0.237) and suicidal ideation (p = 0.752) outcomes. However, we noticed that Egger’s test and funnel plots indicated the existence of publication bias for depression (p = 0.029) and anxiety (p = 0.003) outcomes (Figure 6).

Discussion

This comprehensive review and meta-analysis showed that stress, depression, anxiety, and suicidal ideation were considerably more frequent in individuals with AD than in healthy ones.

Studies have found pathophysiological connections between stress and allergic illness [55]. Several studies have demonstrated that AD patients also have a history of chronic stress and suffer from severe QoL impairment, resulting in significant emotional discomfort. Kwak and Kim showed that individuals with AD experienced much more stress than those without it in this situation [41]. In a similar vein, it was discovered that adolescents with AD had a considerably higher risk of experiencing stress than adolescents without AD [43]. Reports suggest that psychological stress can trigger a neuroendocrine response, which can have various effects on the physiology of the skin [56]. Moreover, it has been suggested that psychological stress results in aberrant skin barrier function. Situations of psychological stress induced by AD include difficulty sleeping, stigmatization, social exclusion due to restrictions on participation in outdoor activities, and humiliation associated with the condition [57].

Additionally, stress biomarkers significantly influence the prognosis of AD related to stress and the recommendation of appropriate therapy. Cai et al. identified CgA as a valuable biomarker in AD [58]. It was also demonstrated that psychological stress might alter salivary composition, potentially worsening AD [59].

The symptoms of AD and these psychological stressors resemble a vicious circle. Although the specific mechanism by which stress affects AD is yet unknown, data suggests that stressful situations worsen AD symptoms [60]. Stress causes the release of neuropeptide and neurotrophin from the hypothalamic-pituitary-adrenal axis, which then influences the course and development of AD by causing epidermal barrier dysfunction and a decreased threshold for itching [61].

Our study concluded that AD contributes to the development of depression across all age groups, consistent with previous findings showing a higher frequency of depression among individuals with AD compared to healthy participants [62,63]. Depressive symptoms in certain dermatologic patients have been linked to issues with body image and cosmetic disfigurement. A recent study examining the connection between pruritus and depression in a sample of individuals with pruritic skin illnesses such as psoriasis, AD, and chronic idiopathic urticaria showed that individuals with higher pruritic ratings also had higher depression scores [64].

There were inconsistent relationships between AD and other depression scores, even though substantial correlations existed between AD and clinical depression and self-reported depressive symptoms. Only a small percentage of AD patients experience depression, contributing to this inconsistency. Hence, the mean depression scores for all AD patients decrease when the depression scores in AD patients without depression are set to 0. The Hamilton Depression Rating Scale (HAM-D) and Beck Depression Inventory scores were significantly higher in AD patients. Originally designed as a screening tool, the HAM-D has proven to be valid and reliable and has developed to become one of the most widely used observer-rated depression scales. It can determine both the degree of depression and the response to antidepressant dosage [65,66]. Nevertheless, no study has examined the validity, reliability, and measurement characteristics of several depression scales in AD patients. Interestingly, Ahn et al. and Vittrup et al. did not show a positive association between AD and depression (OR <1) [25,50]. However, it was shown that patients with severe AD had a notably increased frequency of depression. Hence, it is believed that the severity of dermatitis causes depression [63].

Common risk factors for AD and depression, such as low vitamin D levels, stressful situations, air pollution, and obesity, have been shown to worsen these conditions [67]. However, Hsu et al. found no association between age, education level, marital status, overweight status, and psycho-comorbidities among AD patients [68]. Similarly, Slattery et al. demonstrated that demographic and medical history factors such as age, sex, race, ethnicity, socioeconomic status, body mass index, pubertal status, comorbid allergic disorders (including urticaria, allergic rhinitis, and asthma), and use of glucocorticoid medications-did not impact QoL, sleep deprivation, or depressive symptoms among adolescents with AD [69].

In the past, there has been debate concerning the relationship between anxiety and AD. According to multiple studies, there is no discernible difference in anxiety levels between AD patients and healthy controls and no correlation between anxiety and the severity of AD [25,28], which is inconsistent with our findings. However, the results of this meta-analysis align with other research showing that anxiety is more frequent in individuals with AD than in healthy ones [30-32]. A recent comprehensive population-based analysis also found a strong correlation between moderate-to-severe AD and a higher likelihood of anxiety [18].

The neuroimmunological changes caused by atopy eventually have an impact on neural circuitry and specific brain functions involved in emotion regulation and cognition, much like a vicious cycle. That could account for the sequential phenomenon where sadness and anxiety follow AD [30]. However, more investigation is required to comprehend the mechanism connecting AD to depressive and anxiety disorders [30]. According to Chen et al., adolescents with AD showed a five-fold increase in anxiety disorders compared to adults with AD, who had a risk of significant anxiety disorders around three times higher [30]. This result may indicate that AD in adolescents may be more strongly associated with anxiety issues. This conclusion may be explained by the fact that anxiety problems often start in childhood and appear before depressive disorders [70].

The physical and mental toll of AD may be responsible for the reported elevated risk of suicidal ideation. Individuals with unmanaged AD may experience skin pain, burning, and crippling pruritis [13]. Additionally, it has been shown that AD patients who experience sleep disruption due to pruritus are more likely to harbor suicidal thoughts [71]. The greater likelihood of suicide ideation reported in AD patients could be attributed to psychosocial factors such as the disease’s stigma and shame as well as poor performance at school or work [72]. Research comparing individuals with moderate-to-severe AD to those with mild AD revealed that the latter group had a greater frequency of suicidal ideation but did not carry out actual suicides [18]. More severe AD disease is linked to higher rates of anxiety and depression, severe pruritus, and decreased sleep, all of which may encourage suicidal thoughts [73].

Both biological and psychological factors are likely to contribute to mental health issues related to AD. Pro-inflammatory cytokines, which can cross the blood-brain barrier, are associated with increased AD levels and can trigger central nervous system events such as oxidative stress, neurotransmitter breakdown, altered serotonin metabolism, and reduced neurogenesis in various brain regions. These cytokines are also found at higher levels in the skin and blood. Therefore, excessive inflammation in AD may be linked to depression and suicidality [74].

On the other hand, the scant information on completed suicides revealed contradictory results. Singhal et al. [75] reported a higher suicide risk among AD patients compared to healthy controls, in contrast to Thyssen et al. [18], who observed no difference in the risk of suicide among AD patients compared to healthy controls. These findings could not be reliable due to the wide range of sample sizes. Moreover, there is a lack of published research on suicidal behavior in children and adolescents with skin problems. Employing suitably large sample sizes and tracking them over extended periods may be required to identify variations in suicide rates between individuals with and without AD.

Strengths and limitations

This study analyzed the stress, depression, anxiety, and suicidal ideation outcomes among patients with AD, considering studies from various countries. In this investigation, we searched nine different databases. The key advantages of this paper were the wide scope of the investigations and the large sample size. We also demonstrated the superior quality of the included research, which had a good or acceptable quality grade.

However, this study had some drawbacks and limitations. Firstly, the number of studies examining stress outcomes was limited. Secondly, we observed that approximately 30% of the studies were from the same country (Republic of South Korea). Publication bias probably played a role in the less representative, nonsignificant outcomes because this meta-analysis was based on published data.

Furthermore, the studies that were included in this study used a variety of criteria to define the outcomes, and the majority of them relied on self-reported psychiatric symptoms or a score that had been computed using different measures. Only three studies used a clinical diagnosis to describe outcomes [18,26,30]. Therefore, this meta-analysis analyzed articles that used broadly varying measurement systems of outcomes. Additionally, study demographics and the severity of AD varied. Only studies written in English were included. It is impossible to demonstrate a causal relationship using observational data.

AD has been linked to all concurrent conditions associated with anxiety and depression, including asthma and allergic rhinitis [76]. Thus, it is plausible that these comorbidities could help explain why AD and psychiatric symptoms have a favorable link. These limitations made it impossible to compare the findings of various studies, which contributed significantly to the inconsistencies in this meta-analysis.

## Conclusions

This systemic review and meta-analysis identified a statistically significant association between stress, depression, anxiety, and suicidal thoughts in AD patients, independent of age or gender. Although the criteria used by the chosen research to determine these indicators varied, the concluding results were essentially the same. The dermatologist must take significant steps to promote the mental health and QoL of AD patients in all age groups and genders while also being attentive to the disease’s impact on their mental health. More longitudinal research is required to understand the intricate connections between AD and stress, depression, anxiety, and suicidal ideation, as well as the best ways to prevent and treat them.
